# The impact of bundled payment on the economic burden and satisfaction of patients in Close-Knit County Medical Community in China

**DOI:** 10.3389/fpubh.2025.1530176

**Published:** 2025-04-28

**Authors:** Yanhong Guo, Jialin Qian, Xin Li, Jian Wang, Liangying Zhu, Erdan Huang, Yanchun Zhang, Sheng Nong

**Affiliations:** ^1^School of Public Health, Youjiang Medical University for Nationalities, Guangxi, China; ^2^Health Development Research Center of the National Health Commission, Beijing, China

**Keywords:** medical insurance bundled payment, Close-Knit County Medical Community, economic burden, satisfaction, economic incentive

## Abstract

**Background:**

China is setting up a Close-Knit County Medical Community (CCMC) to connect county hospitals, township health centers, and village clinics. The medical insurance agency will count the number of insured people in the CCMC area and distribute funds to the community as a whole. Then, the county hospital will work with local health facilities to decide how to use these funds. This reform aims to improve primary healthcare services, lower medical costs, support residents’ health, and boost their satisfaction with healthcare.

**Methods:**

This study looked at data from counties in China monitored by County Medical Communities from 2018 to 2022. We used difference-in-differences (DID) to analyze how bundled payments affected patients’ financial burdens, the income of healthcare providers and the satisfaction of patients and healthcare providers.

**Results:**

The bundled payment policy had no significant effect on the average cost per discharged patient (*p* > 0.05). In 2022, the average outpatient expenses increased by 17.58 yuan (*p* < 0.05), while in 2021, the actual reimbursement rates for hospitalization costs rose by 2.18% (*p* < 0.05). The policy also significantly increased the per-capita income of providers in county hospitals and primary care institutions in 2021 (*p* < 0.01); however, we cannot quantitatively isolate the precise marginal contribution of the bundled payment policy to the observed income increases. Additionally, it had no significant impact on the satisfaction levels of either patients or healthcare providers (*p* > 0.05).

**Conclusion:**

Bundled medical insurance payments in the CCMC do not add financial stress for patients and help low-income families. They also boost the income of healthcare providers. However, there is still a need for improvements to enhance overall satisfaction with the healthcare system.

## 1 Introduction

The World Health Organization (WHO) emphasizes the importance of a balanced healthcare structure and equitable resource distribution to enhance health outcomes. According to the World Health Organization (WHO), China’s healthcare accessibility index ranks below the global average, with rural residents facing particularly limited access to quality care (1). Data from the National Health Commission of China reveals that the utilization rate of primary healthcare services remains relatively low, while the tertiary hospitals are overwhelmed with patients, including those with minor conditions that could be managed at the primary level. This not only strains the tertiary care system but also increases healthcare costs. According to the World Health Organization’s Global Health Expenditure Database, China’s out-of-pocket (OOP) payments accounted for 35.3% of total health expenditure in 2019—significantly higher than the OECD average of 20.1% (2).

To tackle these issues and shift focus toward primary care, the General Office of the State Council issued the “Guiding Opinions on Promoting the Construction and Development of Medical Consortiums” [Guo Ban Fa(2017) No. 32] on April 23, 2017. The document suggests creating medical groups across the country, focusing on forming Close-Knit County Medical Communities (CCMCs). These communities will center around county hospitals and connect to township health centers and village clinics. The aim is to encourage collaboration among different medical facilities in the county and to improve the efficiency of medical services. In August 2018, the National Health and Family Planning Commission and the State Administration of Traditional Chinese Medicine issued a notice about improving the hierarchical medical system [National Health First (2018)28]. This notice focused on implementing bundled payments for CCMC. This payment model encourages county hospitals to focus on treating more complex and severe cases, while patients with less serious conditions can be referred to primary healthcare facilities like township health centers and village clinics. Primary healthcare institutions are expected to lower the costs of serious illnesses and injuries by offering preventive care and health management, as well as reducing the number of patients who need to be re-hospitalized for chronic diseases. As a result, residents in these communities can receive continuous and comprehensive healthcare services, which can improve their overall health. This can help create a cycle in which the healthier the residents who sign up, the more the Medicare fund has left over, and the more revenue the healthcare providers receives (The rest of the medical insurance fund belongs to the healthcare providers in the CCMC).

International experience with similar payment reforms offers mixed insights: A study found that compared to fee-for-service payment, bundled payments can help slow the growth of payer spending (3). A study conducted by New York University Langone Medical Center on the impact of bundled payments on fund balances revealed: After implementing bundled payments, medical insurance expenditures for knee replacement surgery decreased by $3,017, expenditures for heart valve surgery were reduced by $2,999, while expenditures for spinal surgery increased by $8,291. Notably, the 30-day readmission rates for all surgical types remained stable, showing no significant changes compared to the pre-reform period (4). However, bundled payment is not without flaws. Some scholars have found that healthcare providers may engage in “cream-skimming” behavior—selectively admitting patients with lower medical costs while potentially compromising equal access to care for patients requiring higher-cost treatments (5, 6). Other research has revealed that although initial hospitalization costs decrease under bundled payment, providers aiming to control total episode costs tend to reduce post-acute care services within the 90-day period, which subsequently leads to increased home healthcare expenditures for patients during later stages of recovery (7). Chinese research on bundled medical insurance payments within the CCMC has primarily focused on qualitative studies (8–10), including experiences from pilot counties, the functioning of incentives, and the challenges faced (10–13). Despite these efforts, there remains a significant gap in empirical analysis regarding how bundled payments affect patients’ financial burdens, their satisfaction, and providers’ income.

This study employs a difference-in-differences approach to systematically evaluate the effects of bundled payments in CCMC, with three primary objectives: First, assessing the reform’s impact on reducing patients’ financial burdens. Second examining changes in healthcare providers’ income levels. Third, measuring improvements in satisfaction among both patient and healthcare providers. Additionally, this study will combine empirical analysis to determine whether healthcare providers have demonstrated any unexpected behavioral changes under the bundled payment system. These empirical findings will provide critical insights into current implementation challenges of CCMC bundled payment reforms, offering evidence-based policy recommendations to optimize China’s healthcare payment system.

## 2 Theoretical framework

The bundled payment reform in CCMCs was theoretically designed to establish a virtuous cycle where healthier enrolled populations generate greater medical insurance fund surpluses, thereby increasing provider revenues. However, during policy implementation, inherent information asymmetry between healthcare providers and patients—with providers holding superior clinical knowledge—enables them to predict treatment costs and resource utilization in advance. This informational advantage may induce moral hazard (14), as providers could potentially manipulate service provision to maximize fund surpluses at the expense of optimal care delivery.

**Figure 1:**
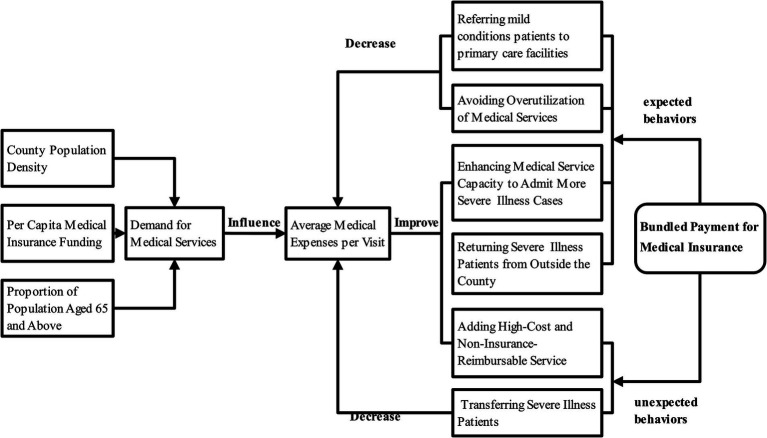
The impact pathway of bundled payment under the medical insurance scheme on the average medical expenses per capita of county-level insured individuals.

This study proposes some essential ideas about how medical expenses change when bundled payment for medical insurance is used within the CCMC. There are three possible outcomes for patients: their expenses might “increase,” “decrease,” or “stay the same” (Figure 1). Factors that are hard to control, population density in a county, the amount of money available for medical insurance per person, and the percentage of people over 65, can affect the demand for medical services. This demand can then impact the prices of those services. For example, when people have more money to spend on health insurance, they can usually afford better healthcare. In counties with a larger older adult population, there tend to be more residents dealing with chronic and basic health issues. Additionally, high population density in a county often leads to an increased demand for medical services.

After CCMC introduced the Medicare bundled payment system, healthcare providers showed two types of behavior: “expected” and “unexpected.”

“Expected behaviors” include:

- Referring mild conditions patients to primary medical institution.
- Avoiding unnecessary medical services.
- Improving their own ability to provide medical services.
- Admitting more patients with serious illnesses.

“Unexpected behaviors” involve:

- Refusing to treat seriously ill patients, shifting medical costs to patients to save on bundled insurance funds.
- Offering expensive treatments not covered by insurance.

These behaviors can influence both outpatient and inpatient per-capita expenditure. Empirical evidence suggests that patients typically incur higher medical costs when seeking care at advanced healthcare institutions outside the county compared to receiving treatment within CCMC (15). Within this context, provider behaviors affecting per-capita costs can be categorized as follows: Appropriate cost-reduction measures include referring patients with minor conditions to primary medical institutions and avoiding unnecessary medical services to optimize resource allocation; Inappropriate cost-reduction strategies may involve the refusal to treat severe or complex cases to minimize additional medical insurance expenditures. While retaining patients within the county system generally reduces overall costs compared to extra-county treatment, the management of severe cases within the county still incurs significantly higher expenses than routine care. Consequently, appropriate cost-increasing behaviors encompass enhancing institutional capacity to provide medical services, thereby attracting severe cases that would otherwise seek higher-cost treatment outside the county. Conversely, inappropriate cost-increasing practices involve the excessive provision of non-reimbursable premium services, which transfers financial burdens to patients while preserving bundled payment funds.

## 3 Methods

### 3.1 Data sources and sample selection

The research for this study used data from the Performance Assessment and Surveillance System for the CCMC. This system collected monitoring data from over 1,000 pilot counties across the between 2018 and 2022. The Health Development Research Center of the National Health Commission put together the data, which was accessed through an online platform. Due to data confidentiality requirements, this study was conducted under the guidance of China’s National Health Development Research Center, with a random selection of 528 pilot counties from over 1,000 potential candidates serving as the research sample.

### 3.2 Research design

In May 2019, the National Health Commission and the State Administration of Traditional Chinese Medicine released a document called “Notice on Promoting the Construction of Compact County-level Medical and Health Communities” [National Health Letter (2019) No. 121]. The policy documents specify the implementation of bundled medical insurance payments for CCMCs, but considering the inherent time lag in policy execution where effects do not manifest immediately, this study designates 2020 as the formal commencement year of the bundled payment reform. Accordingly, among the research subjects, pilot counties that implemented the bundled payment policy in 2020 are classified as the treatment group, while non-implementing pilot counties serve as the control group for comparative analysis.

Indicators for gauging the financial burden on patients include the average cost of discharged patients, average cost of outpatient service and the actual reimbursement rates of hospitalization costs; indicators for assessing the income of healthcare providers and the satisfaction levels of both healthcare providers and patients include per capita income of primary medical and health institutions, per capita income of county hospitals, patient satisfaction in the county, and healthcare providers satisfaction in medical community; Additionally, control variables, such as the logarithmic values of each county’s average permanent population and per capita gross domestic product, were included, along with other relevant factors.

### 3.3 Model

This study employs a quasi-experimental difference-in-differences (DID) approach to evaluate the impact of bundled payment reforms implemented in CCMC. The Difference-in-Differences (DID) method is a widely used econometric approach for evaluating the causal effects of policy changes or interventions (16). Its fundamental principle involves estimating the net policy effect by comparing outcome differences between the treatment group (affected by the policy) and the control group (unaffected), both before and after policy implementation (17). The specific calculation process entails: First, measuring the outcome variable change in the treatment group pre- versus post-intervention. Second, measuring the corresponding change in the control group. Third, subtracting the control group’s change from the treatment group’s change to isolate the policy’s net effect (18). DID’s primary advantage lies in its ability to control for time-invariant unobserved factors through its dual differencing mechanism, thereby enhancing result reliability. However, this method has several limitations: it may produce biased estimates when treatment effects vary over time and it also faces challenges in addressing spillover effects between groups. Nevertheless, this method remains widely employed in healthcare research to evaluate the impacts of policy interventions, medical treatments, or health service reforms on various outcomes, including patient health status (19), healthcare utilization (20, 21), and medical expenditures (22).

The research design is structured as follows:


$$Y_{\mathrm{it}}=\alpha_{0}+did_{t}D_{i}T_{t}+year_{t}T_{t}+\beta X_{\mathrm{it}}+\mu +\varepsilon_{\mathrm{it}}$$

$Y_{\mathrm{it}}$ is the dependent variable; $\alpha_{0}$ is the constant term; $D_{i}$ is the binary variable indicating whether to implement bundled payment; $T_{t}$ is the year vector including 2018–2022, $year_{t}$ is its coefficient, namely *year_2019_*, *year_2020_*, *year_2021_*, and *year_2022_*, indicating the annual changes in the dependent variable relative to the base year 2018; $D_{i}T_{t}$ represents the interaction term between “year variables” and “implementation of bundled payment,” with coefficients $did_{t}$ indicating the net effect of bundled payment on the dependent variable for each year, including *did_2018_*, *did_2019_*, *did_2021_*, and *did_2022_*, they represent the net effects of the policy in 2018, 2019, 2021, and 2022 relative to the base year 2020; Since bundled payment policies were not implemented in 2018 and 2019, *did_2018_* and *did_2019_* represent “blank analyses,” indicating the calculated policy impact even without bundled payment implementation, theoretically lacking statistical significance. They show that the trend of change in the dependent variable between the experimental and control groups before bundled payment implementation is the same, i.e., the parallel trend test. If the difference is not statistically significant, it means that the parallel trend test is satisfied; if there is statistical significance, it indicates the presence of systematic differences between the experimental and control groups before policy implementation, and the subsequent changes may not necessarily be the effect of the policy; $X_{\mathrm{it}}$ represents various control variables affecting the dependent variable; β is the coefficient vector of control variables; μ represents individual fixed effects that do not change over time; $\varepsilon_{\mathrm{it}}$ is the random error term. All analyses were conducted using the Stata 15.0 software, with statistical significance indicated by *p <* 0.05.

## 4 Results

### 4.1 Basic variable description

We select the average values of various factors in three essential years: before the policy was implemented (2018), 1 year after it was implemented (2020), and 3 years later (2022) (Table 1). We performed independent samples t-tests to compare variable means between the treatment and control groups, and also compared the treatment group’s 2022 values with their baseline values.

**Table 1:** Basic conditions of each variable.

	2018		2020		2022	
Variables	Experimental group	Control group	Experimental group	Control group	Experimental group	Control group
Average cost of discharged patients (¥)	7098.62	6722.78	8005.43^*^	7740.80	8426.69	7616.44
Average cost of outpatient service (¥)	134.75	141.17	162.93	164.93	164.89^#^	157.44
Actual reimbursement rates of hospitalization costs (%)	61.36	61.16	63.16^*^	61.69	65.09^#^	64.72
Per capita income of county hospitals (¥)	226552.00	223302.10	266702.30	260953.10	290410.9^*#^	263108.80
Per capita income of primary medical and health institutions (¥)	109982.20	114305.10	137326.10	126396.30	180914.4^*#^	136568.30
Patient Satisfaction in the County	76.15	75.26	90.98^*^	82.20	92.54^*#^	89.15
healthcare providers satisfaction in Medical Community	72.79	71.80	89.31^*^	81.35	91.71^*#^	86.25
County population density	454.07^*^	696.86	504.85^*^	791.48	633.95^*^	561.33
The proportion of people over 65 years old in the resident population of a county	12.33	11.95	12.85	13.05	13.67^*^	12.39
Ln (GDP per capital)	1.49	1.66	1.62	1.88	2.01	1.98

^*^Indicates that the difference between the experimental group and the control group in the current year was statistically significant (*p* < 0.05). ^#^Indicates that the difference between the experimental group and the baseline value in 2018 is statistically significant (*p* < 0.05).

In 2018, right before the policy started, the only significant difference we found was that the county population density in the experimental group was lower than in the control group (*p <* 0.05). Most other factors did not show significant differences (*p* > 0.05). After the policy on medical insurance payments was put in place in 2020, several necessary measures in the experimental group showed significant improvements compared to the control group. These included average cost of discharged patients, actual reimbursement rates of hospitalization costs, patient satisfaction in the county and healthcare providers satisfaction in medical community (all *p <* 0.05). By 2022, the bundled payment group demonstrated significantly higher means than both the control group and their own 2018 baseline values in per-capita income of county hospitals, per-capita income of primary medical and health institutions, county patient satisfaction, and healthcare providers satisfaction in medical community (*p <* 0.05). Additionally, the treatment group had significantly higher means than the control group in average cost of outpatient service and actual reimbursement rates of hospitalization costs (*p <* 0.05). Although these results are consistent with what we expected from the bundled payment policy, more analysis is needed to see if the policy directly caused these changes.

### 4.2 Difference-in-differences analysis of bundled payment policy effects

The coefficients *did_2018_* and *did_2019_* represent the estimated net effects of the bundled payment policy on outcome variables in 2018 and 2019, respectively. Theoretically, these coefficients should be statistically insignificant, so these coefficients essentially serve as a parallel trends test between treatment and control groups. If statistically significant effects were observed in these pre-intervention periods, it would indicate the existence of pre-existing differential trends between the bundled payment and non-bundled payment groups prior to policy implementation. In such cases, post-intervention outcome changes could not be entirely attributed to the policy effect in the difference-in-differences analysis.

As shown in Table 2, the *did_2018_* and *did_2019_* estimates for average hospitalization costs, per-capita outpatient expenditures, and actual inpatient reimbursement rates all showed statistically non-significant differences (*p* > 0.05). This indicates that: these three outcome variables exhibited no significant divergence prior to the 2020 policy implementation, and their temporal trends remained parallel during both the 2018–2019 and 2019–2020 periods. The parallel trends assumption holds even after accounting for potential confounding factors, as evidenced by the consistent non-significance of pre-treatment coefficients across all specifications. Consequently, the difference-in-differences estimates of bundled payments’ impact on patient financial burden demonstrate robust causal identification free from pre-existing differential trends.

**Table 2:** The net effect of bundled payment on patients’ financial burden.

Independent variable	Average cost per discharge patient (¥)	Average cost of outpatient service (¥)	Actual reimbursement rates of per discharge patient (%)
*did_2018_*	−2,048.437	−2.851	−0.784
	(2,312.758)	(4.208)	(0.691)
*did_2019_*	396.653	−2.117	−0.418
	(615.932)	(4.160)	(0.664)
*did_2021_*	−381.892	0.530	2.184^**^
	(890.730)	(5.431)	(0.879)
*did_2022_*	−461.039	17.576^**^	1.004
	(912.136)	(8.431)	(1.035)
*year_2019_*	−2,410.261	12.189^***^	0.123
	(2,175.895)	(3.153)	(0.459)
*year_2020_*	−2,184.374	23.796^***^	0.796
	(2,173.999)	(3.721)	(0.545)
*year_2021_*	−1,596.919	30.156^***^	0.941
	(1,757.092)	(6.261)	(0.909)
*year_2022_*	−1,544.3480	7.712	2.537^***^
	(1,918.712)	(8.820)	(0.976)
County population density	−0.126^***^	0.000	−0.000
	(0.004)	(0.000)	(0.000)
The proportion of people over 65 years old in the resident population of a county	0.025	0.000	−0.023^***^
	(0.063)	(0.002)	(0.004)

Robust standard errors in parentheses ^*^P < 0.1, ^**^P < 0.05, ^***^P < 0.01.

Table 2 demonstrates the *did_2021_* and *did_2022_* estimates for the average cost of discharged patients showed statistically non-significant effects (both *p* > 0.05), indicating that the bundled payment policy had no measurable impact on the average cost of discharged patients in either 2021 and 2022. For average cost of outpatient service, we observed a significant positive policy effect in 2022 (*did_2022_* = 17.576 yuan, *p* < 0.05), accompanied by progressively increasing temporal effects (*year_2019_* = 12.189 yuan; *year_2020_* = 23.796 yuan; *year_2021_* = 30.156 yuan; all *p* < 0.01). The analysis of actual reimbursement rates of hospitalization costs revealed a significant positive policy effect in 2021 (*did_2021_* = 2.184%, *p* < 0.05) and a significant temporal improvement in 2022 (*year_2022_* = 2.537%, *p* < 0.01), with no other years showing statistically significant effects (all *p* > 0.05). Regarding control variables, higher county population density was significantly associated with reduced average hospitalization costs (*p* < 0.01), while an increased proportion of older adult residents (≥65 years) correlated with lower inpatient reimbursement rates (*p* < 0.01), all other control variables showed non-significant associations.

Table 3 reveals for per-capita income at both county hospitals and primary medical and health institutions, the *did_2018_* and *did_2019_* estimates showed statistically significant differences (both *p* < 0.01), indicating violation of the parallel trends assumption. Specifically, during the pre-intervention periods (2018–2019 and 2019–2020), the treatment group’s per-capita income of county hospitals exhibited 2.294 and 2.170% faster growth rates, respectively, compared to controls, while primary institutions showed 0.406 and 0.402% faster growth. These pre-existing differential trends suggest that post-policy income changes cannot be fully attributed to the bundled payment reform, with confounding factors like economic growth or compensation policies contributing to the observed effects. Conversely, patient satisfaction in the county and healthcare providers satisfaction in medical community demonstrated non-significant pre-intervention differences (both *p* > 0.05), confirming parallel trends assumption.

**Table 3:** Net effect of package payment on medical staff income and doctor-patient satisfaction.

Independent variable	Ln [per capita income of county hospitals (¥)]	Ln [per capita income of primary medical and health organizations (¥)]	Patient satisfaction in the county	Healthcare providers satisfaction in medical community
*did_2018_*	2.294^***^	0.406^***^	−7.035	−7.643
	(0.163)	(0.043)	(4.983)	(5.580)
*did_2019_*	2.170^***^	0.402^***^	0.283	0.027
	(0.162)	(0.042)	(4.832)	(5.266)
*did_2021_*	2.134^***^	2.019^***^	−4.790	−4.236
	(0.154)	(0.321)	(13.438)	(14.464)
*did_2022_*	0.038	0.148^*^	−5.931^*^	−3.511
	(0.168)	(0.089)	(3.478)	(3.830)
*year_2019_*	0.168	0.099^***^	2.723^***^	3.787^***^
	(0.121)	(0.015)	(0.933)	(0.896)
*year_2020_*	2.458^***^	0.655^***^	7.281^**^	8.918^***^
	(0.167)	(0.048)	(2.930)	(3.172)
*year_2021_*	0.347^*^	−1.195^***^	15.664^***^	18.061^***^
	(0.180)	(0.325)	(2.411)	(2.580)
*year_2022_*	2.435^***^	0.698^***^	16.762^***^	16.644^***^
	(0.154)	(0.079)	(2.886)	(3.197)

Robust standard errors in parentheses ^*^P < 0.1, ^**^P < 0.05, ^***^P < 0.01.

The temporal trend analysis reveals from 2018 to 2022, the per capita income for county hospitals showed a slow increase (*year_2019_* = 0.168; *year_2020_* = 2.458, *p* < 0.01; *year_2021_* = 0.347, *p* < 0.1; *year_2022_* = 2.435, *p* < 0.01), while that per capita income for primary medical and health institutions first declined and then rose (*year_2019_* = 0.099; *year_2020_* = 0.655; *year_2021_* = −1.195; *year_2022_* = 0.698; all *p* < 0.01). The bundled payment policy significantly increased per-capita income for both county hospital providers (*did_2021_* = 2.134%, *p* < 0.01) and primary medical and health institutions (*did_2021_* = 2.109%, *p* < 0.01). Notably, the policy effect at medical and health institutions offset the negative temporal trend observed that year (*year_2021_* = −1.195, *p* < 0.01). However, since there were significant differences between the experimental and control groups in 2018 and 2019, we cannot completely attribute the increased effects seen in 2021 to the policy alone. Specifically, we cannot quantitatively isolate the precise marginal contribution of the bundled payment policy to the observed income increases.

For healthcare providers satisfaction in medical community, the policy effects (*did_2021_* and *did_2022_*) showed no statistically significant impact (both *p* > 0.05), indicating that despite observed income increases at both county hospitals and primary medical and health institutions, providers satisfaction levels remained unchanged. Patient satisfaction in the county demonstrated a declining trend post-intervention, with negative did coefficients in both 2021 and 2022 (the latter significant at 10% level), potentially attributable to restrictions on patient flow implemented before the COVID-19 transition. Temporal trends exerted significant influence on both satisfaction measures: patient satisfaction coefficients increased progressively from 2.723 (2019) to 16.762 (2022) (all *p* < 0.01), while provider satisfaction showed similar annual growth from 3.787 (2019) to 16.644 (2022) (all *p* < 0.01), indicate that both patient satisfaction and healthcare providers satisfaction have shown year-on-year improvement.

## 5 Discussion

### 5.1 Implementation of bundled payment in Close-Knit County Medical Communities can alleviate patients’ economic burden to a certain extent

The empirical results demonstrate that while the bundled payment policy showed no statistically significant effect on average cost of discharged patients (*p* > 0.05), the negative coefficient of its difference-in-differences estimate indicate that the bundled payment policy has a certain inhibitory effect on the increase of average cost of discharged patients. Coupled with the policy’s significant positive effect on actual inpatient reimbursement rates in 2021 (*did_2021_* = 2.184%, *p* < 0.05), indicates that the treatment group implementing bundled payments was able to increase patients’ actual inpatient reimbursement rates despite annual growth in average cost of discharged patients, which holds significant importance for alleviating patients’ financial burdens and addressing the issue of high healthcare costs. Rong and Yang (23) and He et al. (24) also obtained consistent results, showing that although per-capita medical costs initially increased after implementation of the bundled payment policy, they exhibited an overall declining trend, while patients’ actual inpatient reimbursement rates significantly increased following the policy’s implementation. Furthermore, Nong et al. argues that given more time for policy implementation, the pro-poor financial relief effects of bundled payment policies in CCMC would become more pronounced (15). Accordingly, this study concludes that the implementation of bundled payment policies in CCMC can effectively reduce patients’ financial burdens to a certain extent.

We also found that an increase in the proportion of the population aged 65 and above within counties leads to higher medical costs, which is consistent with findings from other studies (25, 26). This occurs because older adult individuals generally have poorer physical functioning, suffer from more underlying diseases, require greater medical services, and are characterized by features such as frequent hospital readmissions and prolonged hospital stays. Consequently, when older adult patients are hospitalized due to illness, their incurred medical costs are generally higher, thereby consuming more medical insurance funds. Buchner and Wasem (27) also noted that the growth rate of per capita healthcare expenditures for the older adult outpaces that of younger populations, causing the aggregate medical expenditure curve to become progressively steeper over time. This suggests that county medical communities implementing bundled payments should strengthen health management services for older adult populations by implementing regular health examinations, health consultations, and chronic disease management measures, while enhancing seniors’ health awareness and promoting healthy lifestyle habits to reduce high-cost medical expenditures caused by major illnesses at the source. Additionally, for older adult individuals already suffering from chronic diseases or at high risk of developing them, the role of family doctor teams should be fully utilized to implement personalized health education, scientific outreach, and behavioral intervention programs. This approach can effectively control disease progression in older adult patients with chronic conditions or high-risk profiles, thereby reducing their risks of readmission and premature hospitalization. Through these dual strategies, not only can the financial burden on older adult patients be effectively alleviated, but the utilization efficiency and risk resilience of medical insurance funds can also be enhanced.

### 5.2 The pathways of bundled payment in reducing patients’ economic burden lie in the dual effects

The empirical results demonstrate that the bundled payment policy produced a statistically significant positive effect on actual inpatient reimbursement rates, indicating a genuine increase in patients’ reimbursement levels. This outcome rules out the possibility that healthcare providers increased the provision of non-reimbursable premium services, which would have decreased the actual reimbursement rates. According to official national county medical community survey data, CCMC in 2022 showed a 4.04% increase in the proportion of county-level hospitalizations and a 0.59% rise in intra-county medical insurance fund expenditure rates. These findings corroborate our study’s results demonstrating increased average cost of discharged patients and average cost of outpatient service in county medical community implementing bundled payments, indicating a growing proportion of high-cost patients within county medical community. This indicates that the enhanced service capacity of county hospitals has led patients who previously sought care outside county boundaries to return for treatment within the county. Under the incentive mechanisms of bundled payments, healthcare providers have facilitated the return of more high-cost patients with severe and complex conditions to county hospitals. LU’s study yielded consistent results, demonstrating that pilot counties achieved an 8% increase in intra-county medical insurance fund expenditures and a 15.4% growth in average cost of outpatient service in county medical community compared to non-pilot counties (28).

Under the incentives of the bundled payment policy, CCMC in pilot counties not only retained more patients within county hospitals but also appropriately redirected patients with mild conditions to primary medical and health institutions. This approach effectively reduced unnecessary medical resource consumption through strict enforcement of hospitalization criteria, ensuring that only patients with genuine clinical needs were admitted. Consequently, it achieved medical cost savings while implementing the tiered healthcare system policy, simultaneously increasing actual reimbursement ratio of hospitalization costs and alleviating patients’ financial burdens. Although existing qualitative studies have reached similar conclusions, revealing that the overall service capacity of county-level medical institutions has been enhanced after the reform, with increased rates of medical visits within the county, higher numbers of outpatient and emergency visits at the primary level, reduced per capita patient costs, and increased actual reimbursement ratios for inpatient expenses (29, 30), the current study, by employing quantitative analysis, is capable of quantifying the effectiveness of the reform, thereby enhancing the credibility and persuasiveness of the research findings. However, some studies finding that bundled payment policies primarily encouraged patient visits to county-level hospitals without effectively redirecting patients to primary medical and health institutions (31). This phenomenon stems from the limited service capacity of primary medical and health institutions in those pilot counties, which fell substantially below provincial averages, rendering them unable to meet patient demand. These findings highlight significant regional disparities in the implementation of CCMC reforms across China, with deficiencies in primary medical and health institutions capacity remaining in certain areas.

### 5.3 The incentive effects of bundled payment in medical insurance warrant strengthening, with room for policy improvement

The results show that the bundled payment policy positively affected healthcare providers’ income in 2021. However, this effect cannot be attributed to the medical insurance bundled payment policy because before the implementation of the medical insurance bundled payment policy, there was a significant difference in the income of healthcare providers in the experimental group and the control group, and the effect of the bundled payment policy on the increase of medical staff’s income in 2022 was not significant. In recent years, China has been implementing the reform of the salary system in public hospitals, which is expected to lead to an annual increase in the income of healthcare providers. The positive impact of the bundled payment policy on the income of healthcare providers will also overlap with this, which is one of the reasons why the impact brought by the bundled payment policy in this study could not be identified and quantified separately.

Although satisfaction among healthcare providers and patients has been increasing each year, the impact of the medical insurance bundled payment policy is not significant. Compensation levels represent a key determinant of healthcare providers satisfaction (32). While the bundled payment policy was originally designed to allocate medical insurance fund surpluses as performance incentives for healthcare providers, however, there are still disagreements among different departments regarding the understanding of the reward for the surplus of medical insurance funds. Moreover, some medical communities have not yet generated a surplus of medical insurance funds. Both of these factors can, to some extent, weaken the economic incentive effect of the bundled payment policy (33), thereby reducing the satisfaction of healthcare providers. Wang et al. (34) found that in CCMC more than half of the primary medical and health institutions’ healthcare providers believed that their personal performance and income did not increase compared with that before the reform. Chen et al. (35) found that the per capita income distribution between county hospitals and primary medical and health institutions was unfair, which led to low satisfaction among healthcare providers and thus dampened their enthusiasm for participating in the reform. Another critical determinant of provider satisfaction is workload intensity—studies demonstrate that even with higher compensation, excessive workload reduces satisfaction (36). In CCMC, county hospitals assume pivotal roles in resource allocation and technical support for primary medical and health institutions. While our study confirms these county hospitals successfully enhanced primary medical and health institutions’ capacity and appropriately redirected mild cases—thereby improving insurance fund efficiency and reducing patient costs—the expanded responsibilities have substantially increased their staff workloads. Crucially, the magnitude of income growth often fails to match either the additional workload or effort expenditure, exacerbating provider dissatisfaction. To enhance medical providers satisfaction, policymakers should refine internal evaluation mechanisms by developing quantifiable metrics that accurately assess each member institution’s contribution to surplus generation, which should then inform equitable surplus distribution rules to ensure fair compensation aligned with actual performance.

A significant factor impacting patient satisfaction is the cost of medical care. Although we found that bundled payments have improved actual reimbursement rates of hospitalization, the costs for both outpatient and inpatient services keep going up each year. This increase is driven by factors like inflation, the cost of raw materials, and technological advances (37). Therefore, to enhance patients’ sense of gain and satisfaction, CCMC need to improve relevant measures in the following aspects: Strengthen health management services for contracted residents, develop personalized health services for different groups of contracted residents to improve their quality of life and health levels, prevent serious illnesses and complications, and thereby reduce patients’ economic burdens; Utilize various media platforms to enhance the promotion of close-knit county-level medical communities and related policies, enabling contracted residents to have a deep understanding of these policies and increasing their trust in the medical services provided by county medical communities; Strengthen professional training for healthcare providers within the medical communities to enhance their skill levels and service quality; Establish a feedback system for patients within the medical communities to widely collect suggestions and opinions from patients and make corresponding adjustments and improvements based on patient feedback in a timely manner.

## 6 Conclusion

The implementation of bundled payment in medical insurance within CCMC does not increase the financial burden on patients and has a relatively good poverty-alleviating effect. Under the bundled payment model, the income of healthcare providers within CCMC has also increased. Driven by the economic incentives of bundled payments, patients with severe and major illnesses who originally sought treatment outside the county have returned to seek treatment within the county, which has led to a reasonable increase in the per capita medical costs for patients. To save medical insurance funds, more patients with minor illnesses have been referred to primary-level medical institutions by the medical providers. Healthcare providers have obtained the surplus of medical insurance funds in a reasonable manner, without engaging in negative medical behaviors, such as refusing to treat patients with severe illnesses or providing them with high-priced medical services outside the scope of medical insurance reimbursement. However, the reform has not yet achieved improvements in satisfaction levels among either patients or healthcare providers, indicating that there is still room for optimization and improvement of the policy.

## Data Availability

The original contributions presented in the study are included in the article/supplementary material, further inquiries can be directed to the corresponding authors.
